# Aqueous Humor Apolipoprotein Concentration and Severity of Diabetic Retinopathy in Type 2 Diabetes

**DOI:** 10.1155/2022/2406322

**Published:** 2022-11-10

**Authors:** Lucia Saucedo, Isabel B. Pfister, Christin Schild, Souska Zandi, Justus G. Garweg

**Affiliations:** ^1^Swiss Eye Institute, Rotkreuz, and Retina Clinic, Berner Augenklinik, Bern, Switzerland; ^2^Department of Ophthalmology, Inselspital, Bern University Hospital, University of Bern, Bern, Switzerland

## Abstract

An imbalance of plasma apolipoproteins has been linked to diabetic retinopathy (DR); however, there is scarce information regarding their presence in the aqueous humor (AH) and their role in DR. Here, we aimed at analysing the relationship between apolipoprotein concentrations in human AH and the severity of DR. Concentrations of apolipoproteins were measured retrospectively in patients with type 2 diabetes mellitus (T2DM) without DR (*n* = 23), with mild to moderate nonproliferative DR (NPDR) (*n* = 13), and advanced NPDR/proliferative DR (PDR) (*n* = 14) using a multiplex immunoassay. Compared to the non-apparent DR group, the concentrations of seven apolipoproteins were elevated in advanced NPDR/PDR (Apo AI 5.8-fold, Apo AII 4.5-fold, Apo CI 3.3-fold, Apo CIII 6.8-fold, Apo D 3.3-fold, Apo E 2.4-fold, and Apo H 6.6-fold). No significant differences were observed in apolipoprotein concentrations between patients with non-apparent DR and healthy controls (*n* = 17). In conclusion, the AH concentrations of apolipoproteins AI, AII, CI, CIII, D, E, and H increased in advancing stages of DR, suggesting their role in the pathogenesis of DR, which deserves further examination.

## 1. Introduction

Diabetic retinopathy (DR) is the leading cause for vision loss in the working-age population [1]. Classically considered a microvascular disease, growing evidence suggests that neurodegeneration also occurs in the diabetic retina, even prior to visible microvascular changes [2, 3].

Apolipoproteins are important structural components of plasma lipoproteins [4]. Besides their involvement in the formation of lipoproteins and their structural role, apolipoproteins also act as ligands for lipoprotein receptors and serve as activators or inhibitors of the enzymes involved in the metabolism of lipoproteins [5]. Alterations in apolipoprotein expression have been reported in several diseases such as age-related macular degeneration (AMD) [6], in neurodegenerative diseases such as Alzheimer's disease [7] and in vascular disease [8]. Several independent studies have reported a protective effect of apolipoprotein AI (Apo AI) in the development of vascular disease. Apo AI promotes reverse cholesterol transport and prevents LDL oxidation, oxidative stress, inflammation, and endothelial cell dysfunction [9, 10]. It is likely that the lipoprotein and apolipoprotein regulation may be linked to the homeostasis and progression of diabetic retinopathy [11] though evidence is as yet not fully consistent [12]. Part of the reported discrepancy may be explained by differences in the local and systemic regulation in the lipid metabolism [13].

Several epidemiological studies revealed that changes in plasma apolipoprotein concentrations are linked to microvascular pathologies in type 1 and type 2 diabetes mellitus (T2DM) [14–16]. Moreover, an association between serum apolipoprotein levels and DR has been previously reported [17, 18]. Of specific interest, a positive correlation between serum Apo B levels and the severity of DR, and a negative correlation between serum Apo AI levels and the presence of DR were identified [19, 20]. Moreover, increased serum levels of Apo AI have been reported to be protective, while those of Apo CIII and Apo E have been linked to the risk and progression of DR [21]. It has been suggested that Apo AI protective effect in diabetes could be through the activation of AMP-activated protein kinase (AMPK) [22]. The role of Apo AI in angiogenesis has also been described as Apo AI binding protein (AIBP)/high density lipoprotein (HDL)-mediated cholesterol depletion reduces lipid rafts, interferes with vascular endothelial growth factor receptor 2 (VEGFR2) signaling, and inhibits VEGF-induced angiogenesis [23].

Although the involvement of apolipoproteins has been described in several diseases, there barely exists any information regarding the presence and function of apolipoproteins in the healthy human retina and in the pathogenesis of DR. Proteomics studies reveal that Apo AI, Apo AIV, Apo E, and Apo J are the major apolipoproteins in the mouse retina [24]. Apo E knock-out (KO) animals have higher serum and retinal cholesterol concentrations, but their retinae seem normal and show unaltered amplitude of electroretinography (ERG) responses, probably due to compensatory mechanisms [24]. Apo D KO mice, in contrast, show normal levels of retinal sterols, but decreased amplitudes in the ERG, indicating its potential role in retinal function [25]. Furthermore, it has been reported that Apo E interacts with low-density lipoprotein receptor-related protein-1 (LRP-1), a member of the low density lipoprotein receptor family, decreasing retinal vascular abnormalities *in vivo* [26]. Furthermore, lipoproteins containing Apo E protect retinal ganglion cells and Müller cells from N-methyl-d-aspartate (NMDA) -induced excitotoxicity, through a mechanism involving the activation of STAT3 and downregulation of *α*2-macroglobulin [27]. And importantly, these factors might therapeutically be targeted to improve DR progression [28]. Regarding their role in DR, elevated levels of Apo AI and Apo H have been found in the vitreous fluid (VF) of patients with proliferative DR (PDR), in comparison to nondiabetic controls [29], as well as in the neuroretina and retinal pigment epithelium [30]. In another study, an overexpression of Apo AI in the retina of diabetic donors without microvascular abnormalities, but with signs of neurodegeneration, was observed, suggesting that the upregulation of Apo AI might be an early event in the pathogenesis of DR [31]. While this has remained unconfirmed, information is scarce regarding the presence and dynamics of apolipoproteins in the VF and aqueous humor (AH) in the healthy retina and their role in the severity and progression of DR.

Consequently, in this study we aimed at analysing the relationship between apolipoproteins in human AH and the presence and severity of DR. We hypothesize that apolipoprotein levels are altered in early and late stages of the disease. In the present study, we therefore aimed at determining the concentrations of apolipoproteins in the AH of diabetic patients without and with different stages of DR in order to determine stage-specific changes.

## 2. Materials and Methods

### 2.1. Patients

This retrospective study included 50 patients with T2DM, without or with different stages of DR. Samples of AH of patients undergoing cataract surgery were obtained at the beginning of surgery in the Clinic for Vitreoretinal Diseases, Berner Augenklinik, in Bern, Switzerland. Exclusion criteria included type 1 DM, presence of other ocular disease with the exception of cataract, history of any systemic malignant, vascular or inflammatory comorbidity (e.g., rheumatic or autoimmune diseases), presence of a neurodegenerative disease, systemic treatments involving corticosteroids, or immunomodulatory drugs, intravitreal or panretinal laser photocoagulation treatment within six months prior to surgery, vitreous hemorrhage, uveitis, glaucoma, or any concomitant retinal pathology. Additionally, AH samples from 17 healthy controls were also analyzed.

The stage of DR was independently determined by a graduated ophthalmologist blinded to the study protocol prior to the cataract surgery. This determination was made based on the results of dilated stereo biomicroscopy of the anterior and posterior segments of the eye, macular optical coherence tomography (OCT), and wide field fundus images (Optos®), according to the International Clinical Diabetic Retinopathy Disease Severity Scale [32]. Ocular disease was categorized in the following stages: diabetes with non-apparent DR (*n* = 23), mild to moderate NPDR (early DR; *n* = 13), and advanced NPDR/PDR (advanced DR; *n* = 14). Assessment of the DR stage was performed on the same day as collection of the biological samples.

This study was approved by the local Ethics Committee of the University of Bern (Ref. no 152/08) and performed in fully compliance of the tenets of the Declaration of Helsinki (1964). General informed consent was obtained from all study participants before sample extraction for the use of their biological material and clinical data.

### 2.2. Determination of Apolipoprotein Concentration

AH samples (200–250 *μ*l) were collected by anterior chamber tap via aspiration through a 30-gauge needle and stored at -80°C until analysis. The samples were analyzed simultaneously using a multiplex system. Briefly, magnetic microspheres tagged with a fluorescent label and coupled to a specific antibody were added to the samples. Biotinylated detection antibodies and Streptavidin R-Phycoerythrin were added afterwards and the signal was detected by a flow cytometer equipped with two lasers that detect the microsphere type and quantify the amount of bound antigen. Samples were analyzed with the Bio-Plex Pro™ Human Apolipoprotein Assay Panel (Bio-Rad, Hercules, CA, USA), which includes the analytes Apo AI, Apo AII, Apo B, Apo CI, Apo CIII, Apo D, Apo E, Apo H, and Apo J. The plates were read using the Bio-Plex Flexmap 3D system with xPONENT 4.2 software (Bio-Rad, Hercules, CA, USA). All procedures were performed following manufacturer's instructions in a blinded manner by an experienced technician.

### 2.3. Statistical Analysis

Results are expressed as mean ± standard deviation (SD) as well as median and IQR 25–75 (ng/ml), unless stated otherwise [33]. Outliers were identified by a box-plot analysis (box-whisker plot) and extreme outliers (more than three box lengths away from the edge) were excluded from the statistical analysis. Samples ranging below the LLOQ of the assay were replaced by half the value of the LLOQ specific for that protein, as previously established [34].

The Shapiro-Wilk test was applied to determine normal distribution of the data. Since the data did not show a normal distribution, the nonparametric Kruskal-Wallis test was used for intergroup comparison of continuous data and the chi-square test of independence was used to evaluate variables measured at a nominal level. A *p* value <0.05 was considered to be significant (two-sided testing). To control the risk of introducing type I errors as a result of multiple comparisons, we applied the Holm correction, which progressively adapts the threshold for rejecting the null hypothesis. The statistical analyses were performed using the open-source software R (Version 3.3.2 ± 2016 RStudio, Inc.; psych package) and SPSS (version 23.0; IBM SPSS Statistics, Armonk, NY, USA).

## 3. Results

### 3.1. Clinical Characteristics of the Study Population

**Figure 1:**
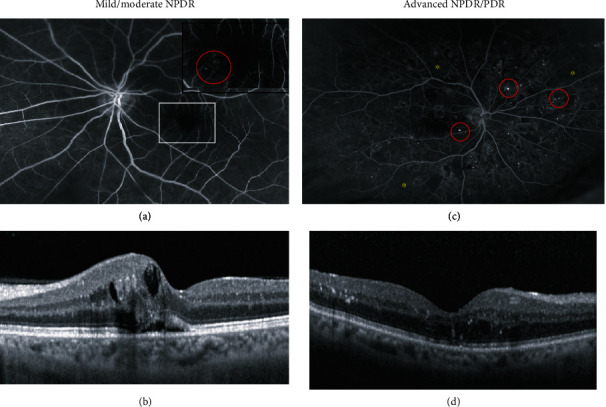
Representative images of two patients with mild NPDR and advanced NPDR: (a) Fluorescence angiography showing discrete changes with single microaneurysms in the macular area and (b) optical coherence tomography image (OCT) of the same patient showing intra- and subretinal fluid leaking from the aneurismatic vessels. The white square in (a) marks the magnified area presented in the upper right corner showing the microaneurysms. (c) Fluorescence angiography and (d) OCT of a patient with advanced NPDR. The red circles in (a) and (c) mark the most prominent of the multiple, throughout the retina scattered microaneurysms. The yellow asterisks in (c) mark areas of ischemia. Corresponding to the central retinal changes visible in the angiography frame, the OCT shows intraretinal fluid and hyperreflective foci probably resembling the leakage of lipids and protein into the retinal tissue.

Diabetic patients were classified into non-apparent DR (no visible diabetic changes), mild/moderate NPDR (single microaneurysms, and spot hemorrhages (Figures 1(a) and 1(b)) and advanced NPDR/PDR (multiple microaneurysms and spot hemorrhages throughout the retina with or without ischemic changes (Figures 1(c) and 1(d)). The clinical information of the patients included in this study is shown in Table 1. According to the patient baseline characteristics, the groups were comparable for age, gender, duration of diabetes, body mass index (BMI), dyslipidemia, hypertension, and medication.

### 3.2. Concentration Levels of Apolipoproteins in DR Stages

**Figure 2:**
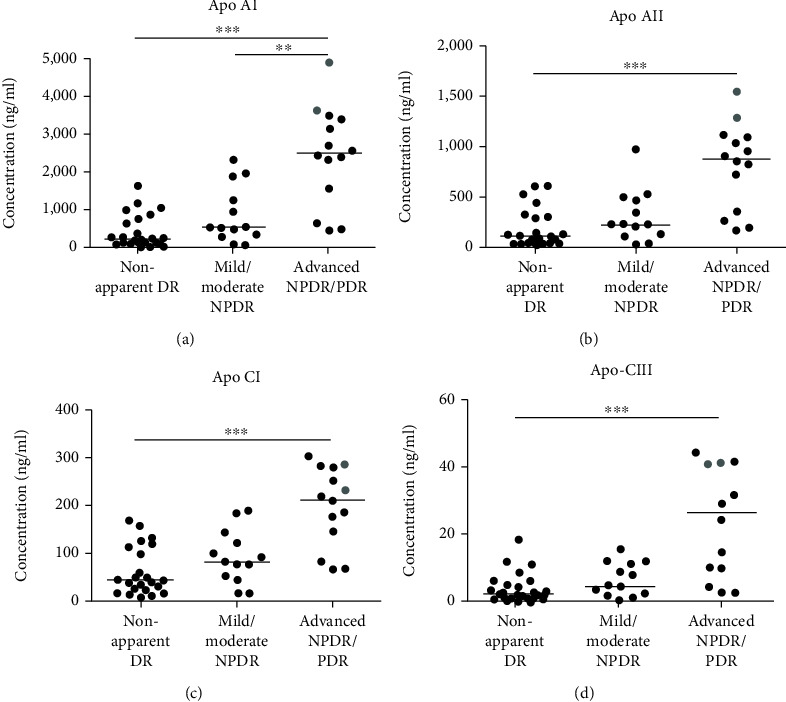
Concentrations of (a) Apo AI, (b) Apo AII, (c) Apo CI, and (d) Apo CIII in the AH of patients with nonapparent DR and patients with different stages of DR. Each point represents data from a single patient. The black lines show the median value. Grey points show the values for two patients with active PDR. ^∗∗^*p* < 0.01, ^∗∗∗^*p* < 0.001. AH: aqueous humor. DR: diabetic retinopathy. NPDR: non-proliferative diabetic retinopathy. PDR: proliferative DR.

**Figure 3:**
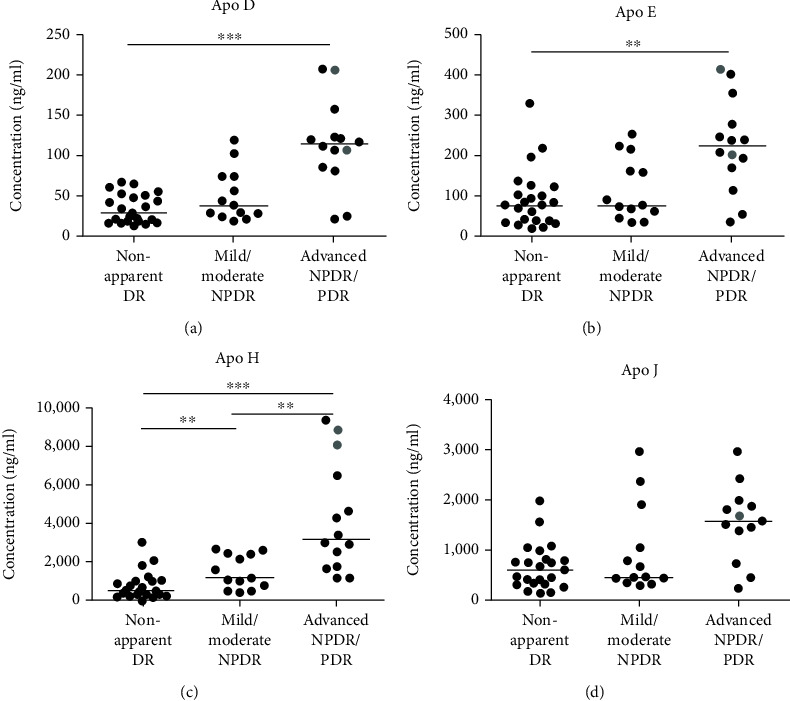
Concentrations of (a) Apo D, (b) Apo E, (c) Apo H, and (d) Apo J in the AH of patients with nonapparent DR and patients with different stages of DR. Each point represents data from a single patient. The black lines show the median value. Grey points show the values for two patients with active PDR, with the exception of Apo J where one patient's value was considered an extreme outlier and excluded from the analysis ^∗∗^*p* < 0.01, ^∗∗∗^*p* < 0.001. AH: aqueous humor. DR: diabetic retinopathy. NPDR: nonproliferative diabetic retinopathy. PDR: proliferative DR.

The concentration of eight apolipoproteins was quantified in the AH of patients with DM with and without DR and the results are shown in Table 2 and Figures 2 and 3. Also, the apolipoprotein concentrations of the patients with mild NPDR and advanced NPDR depicted in Figure 1 are showed in Table 3. All concentrations (with the exception of two) ranged within the detection limits of the assay for the corresponding apolipoproteins, with the exception of Apo B, which was not detected in any of the samples. After application of the Holm correction, increases in the advanced NPDR/PDR group compared to non-apparent DR, for Apo AI (5.8-fold higher), Apo AII (4.5-fold higher), Apo CI (3.3-fold higher), Apo CIII (6.8-fold higher), Apo D (3.3-fold higher), Apo E (2.4-fold higher), and Apo H (6.6-fold higher) were found. In addition, an increase in the AH concentrations of Apo J in the advanced NPDR/PDR group compared to non-apparent DR was also found (2.4-fold higher), but this difference did not remain significant after the Holm correction. Apo H concentrations increased in the mild/moderate NPDR group in comparison to non-apparent DR (2.2-fold higher), as in the advanced NPDR/PDR in comparison to mild/moderate NPDR (2.8-fold higher). In addition, the concentrations of Apo AI were increased in advanced NPDR/PDR compared to mild/moderate NPDR (2.8-fold higher). Finally, increases in the apolipoprotein concentrations in the AH in advanced NPDR/PDR in comparison to mild/moderate NPDR were observed for Apo AII, CI, CIII, D, and E, although these differences were no longer significant after the Holm correction. Two patients in the advanced NPDR/PDR group with active PDR generally showed higher apolipoprotein concentrations in the AH than the remaining patients with advanced NPDR and inactive PDR (Figures 2 and 3).

In addition, the apolipoprotein concentration in the AH of healthy controls was also analyzed and compared to diabetic patients with nonapparent DR. The demographic characteristics of this population and their apolipoprotein concentrations are presented in Table 4. Both populations were similar in age and BMI but differed in gender, dyslipidemia and hypertension. Despite these differences, the concentrations of apolipoproteins between both groups were similar and showed no significant differences.

## 4. Discussion

In the present study, we identified stage-specific increases in the AH concentrations of several apolipoproteins in advanced stages of DR, compared to patients with DM without DR and healthy controls. Our results showed an increase in the AH concentrations of apolipoproteins AI, AII, CI, CIII, D, E, and H in advanced stages of DR compared to non-apparent DR. Interestingly, increased Apo D concentrations have been observed in the aging brain and neurodegenerative pathologies. Since Apo D expression is induced by oxidative stress, acts as an antioxidant preventing lipid peroxidation [35] and has anti-inflammatory potential [36] it was proposed that the induction of Apo D may be part of a basic evolutionary stress response [37]. Moreover, previous studies have reported that lipoproteins containing Apo E protect retinal ganglion cells from apoptosis induced by NMDA and by withdrawal of neurotrophic factors [27, 38].

In the present study, Apo AI and Apo H were found to be two of the most abundant apolipoproteins in the AH and showed significant differences between absence of diabetic retinopathy, mild to moderate NPDR and advanced NPRD/PDR. Our results are consistent with a previous publication which reported that Apo H and Apo AI are the most upregulated apolipoproteins in the VF of patients with PDR compared to healthy controls [29]. In the retina, an antiangiogenic role for Apo H was found *in vitro* and *in vivo,* using a model of oxygen-induced retinopathy indicated by a downregulation of vascular endothelial growth factor (VEGF) and its receptors [39, 40]. Apo AI has anti-inflammatory properties and downregulates retinal inflammation, as has recently been reported [41]. The findings of our study thus confirm published studies. During progression of DR, elevated levels of Apo H and Apo AI might be an attempt to maintain the homeostasis in the diabetic retina, counterbalancing the effects of VEGF and inflammation. Also, Du et al. [42] found the presence of Apo AI in the healthy and DR retina, as well as an increase in the retinal immunofluorescence for Apo B in DR stages [42, 43]. Even though these and the present study observe an increase in apolipoproteins through the development of DR, the source of these proteins is still far from elucidated. A local production of Apo AI has been suggested in the retina [30]. However, it is conceivable that progressive compromise of the blood-retina barrier and/or micro-hemorrhages, which are characteristic of the later stages of DR, contribute to changes in the apolipoprotein concentrations [42, 43]. Determining the source of apolipoproteins is crucial for further elucidating their role in the pathogenesis of DR and might be assessed by the determination of the uveovascular barrier integrity, for example by parallel determination of the AH albumin concentrations in future studies.

Furthermore, the apolipoprotein concentrations in diabetic patients with nonapparent DR were compared to healthy controls. Our results showed that the highest concentrations of apolipoproteins in heathy subjects are Apo H, Apo J, and Apo AI. This result confirms a previous proteomic study in the retinas of C57BL/6 J mice that described Apo AI, Apo AIV, Apo J, and Apo E as the major apolipoproteins [24]. Even though both groups differed in demographic characteristics such as gender and presence of dyslipidemia and hypertension, the concentration of AH apolipoproteins was similar, suggesting that there are no changes in the AH apolipoprotein concentrations in the absence of DR. The relatively high standard deviation observed in these populations might indicate that individual factors beyond local regulation must be influencing the AH apolipoprotein concentration. The different frequencies of hypertension and dyslipidemia between our groups was expected since dyslipidemia and hypertension are highly prevalent in patients with type 2 DM, with a prevalence of 63% and 59%, respectively [44, 45]. Differences in serum levels of apolipoproteins in hypertensive patients vs. healthy controls have been reported [46, 47]. However, there is no evidence of their impact in the AH apolipoprotein concentration. In the present study, the concentration of apolipoproteins in the VF was not determined. Several previous studies observed the presence of Apo AI, Apo H, and Apo J in the VF of diabetic patients and reported their association with PDR and diabetic macular edema [29, 48, 49]. More recent studies have also reported the presence of Apo AII, Apo AIV, Apo CI, and Apo E [50, 51]. Determining the apolipoprotein levels in AH and VF in parallel and calculating their correlation might be of interest to estimate the site of their production as well as their variability and will be addressed in future studies.

The present study was focused on determining the differences in apolipoprotein levels on a local level and therefore, serum apolipoprotein concentrations and serum lipids were not measured. Hence the retinal local regulation is probably driven by the retinal glia, i.e. Müller cells [52] and thus likely independent of the systemic lipid metabolism [53], the impact of local apolipoprotein levels in the different stages of DR may well be assessed on a local level.

Among the strengths of this study, we refer to a standardized DR severity assessment [54] based on clinical and angiographic findings, adding to the homogeneity of the study groups. This was achieved in the advanced NPDR/PDR group by the exclusion of patients with past or active vitreal hemorrhage, intravitreal treatment or panretinal laser photocoagulation within six months prior to inclusion into the study. The significant impact of DR activity is supported by two samples from patients with active PDR without hemorrhage which generally showed higher apolipoprotein concentrations than patients with advanced NPDR and non-active PDR (Figures 2 and 3). The limited number of samples per group does not allow final conclusions to be drawn, but it obviously indicates a link between apolipoproteins and active PDR, which deserves further attention. While the sample size used in this study is relatively small, it is similar to the sample sizes used in comparable studies evaluating AH biomarkers using multiplex systems [55–57] and it allows indicating a potential role of apolipoproteins in the pathogenesis of DR. As previously mentioned, several apolipoproteins have been identified as contributors to the local regulation of oxidative stress, inflammation and angiogenesis [35, 40]. Future in vitro and in vivo studies will allow to distinguish the individual role of each apolipoprotein, as well as the cellular pathways involved, which would help to elucidate their potential beneficial/pathogenic role and open the floor for potential therapies in DR.

## 5. Conclusions

In conclusion, we found that the AH concentrations of apolipoproteins AI, AII, CI, CIII, D, E, and H to be increasing with the progress of DR compared to type 2 DM without DR and healthy controls, suggesting their potential role in the pathogenesis of this disease, which deserves further examination. From a pathophysiological perspective, the upregulation of Apolipoprotein AI may be an attempt to downregulate inflammation, while that of Apolipoprotein H could aim at compensating the overexpression of vascular endothelial growth factor.

## Figures and Tables

**Table 1:** Clinical characteristics of the diabetic population.

	Nonapparent DR (*n* = 23)	Mild/moderate NPDR (*n* = 13)	Advanced NPDR/PDR (*n* = 14)	*p* value
Age (years; mean ± SD)	71.6 ± 9.6	72.1 ± 7.9	69.1 ± 9.5	0.77
Gender F *n* (%)	11 (48)	6 (46)	7 (50)	0.98
Known duration of diabetes (years; mean ± SD)	15.0 ± 12.9	14.0 ± 5.1	17.1 ± 10.4	0.49
BMI (mean ± SD)	28.8 ± 5.6	29.4 ± 5.5	32.0 ± 8.3	0.79
Dyslipidemia *n* (%)	15 (65)	8 (62)	7 (50)	0.55
Hypertension *n* (%)	17 (74)	9 (70)	11 (79)	0.83
*Medication:n(%)*				
Insulin	8 (35)	8 (62)	10 (71)	0.09
Metformin	15 (65)	7 (54)	7 (50)	0.62
Statin	16 (70)	8 (62)	6 (43)	0.32
Fibrate	1 (4)	1 (8)	0 (0)	0.59

Abbreviations: DR: diabetic retinopathy; NPDR: non-proliferative diabetic retinopathy; PDR: proliferative DR; SD: standard deviation; F: female; BMI: body mass index.

**Table 2:** Concentration levels (mean ± SD (median; interquartile ranges (IQR) 25–75); ng/ml) of apolipoproteins in the AH diabetic patients without and with different stages of DR.

	Non-apparent DR (*n* = 23)	Mild/moderate NPDR (*n* = 13)	Advanced NPDR/PDR (*n* = 14)	*p*1	*p*2
Apo AI	421 ± 446 (235; 93-801)	855 ± 761 (543; 288-1572)	2427 ± 1288 (2494; 1328-3393)	<0.0001^∗^	0.0015^∗^
Apo AII	178 ± 191 (104; 40.1-293)	299 ± 262 (221; 106-477)	802 ± 427 (870; 320-1092)	<0.0001^∗^	0.0058
Apo CI	60 ± 49 (42; 22-108)	89 ± 55 (80; 46-129)	195 ± 81 (210; 126-275)	<0.0001^∗^	0.005
Apo CIII	4.0 ± 4.5 (2.2; 1.0-5.8)	6.4 ± 4.9 (4.6; 1.9-11.3)	27.3 ± 23.5 (26.3; 8.3-40.8)	0.00027^∗^	0.0098
Apo D	34 ± 17 (29; 17-50)	51 ± 32 (39;26-74)	114 ± 54 (114; 84-132)	0.00011^∗^	0.0046
Apo E	95 ± 73 (79; 41-127)	118 ± 76 (82; 57-188)	226 ± 113 (226;155-296)	0.0023^∗^	0.0326
Apo H	664 ± 539 (484; 275-943)	1475 ± 885 (1238; 569-2434)	4223 ± 2836 (3177; 1761-6906)	<0.0001^∗^	0.0015^∗^
Apo J	640 ± 452 (564; 302-805)	945 ± 885 (465; 366-1468)	1520 ± 759 (1548; 1010-1925)	0.00526	0.1280

Abbreviations: AH: aqueous humor; *p*1: *p* value between nonapparent DR and advanced NPDR/PDR. *p*2: *p* value between mild/moderate NPDR and advanced NPDR/PDR. ^∗^*p* value remained significant after application of the Holm correction.

**Table 3:** Concentration levels (ng/ml) of apolipoproteins in the AH of the two diabetic patients presented in Figure 1 with mild (a and b) and advanced NPDR (c and d).

AH concentrations (ng/ml)	Apo AI	Apo AII	Apo CI	Apo CIII	Apo D	Apo E	Apo H	Apo J
Mild NPDR (Figure 1(a) and 1(b))	52.6	25.9	15.9	1.2	19.3	37.6	439.4	335.5
Advanced NPDR (Figure 1(c) and 1(d))	2696.7	811.2	249.1	88.6	85.7	278.6	2894.2	1548.6

Abbreviations: AH: aqueous humor; NPDR: nonproliferative diabetic retinopathy.

**Table 4:** Clinical characteristics and concentrations of Apolipoproteins in the AH of healthy controls and diabetic patients with non-apparent DR.

	Healthy controls^1^ (*n* = 17)	No apparent DR (*n* = 23)	*p*-value
Age (years; mean ± SD)	64.6 ± 10.8	71.6 ± 9.6	0.34
*Gender:*			
Females: *n* (%)	16 (94)	11 (48)	0.002^∗^
Duration of diabetes (years; mean ± SD)	N/A	15.0 ± 12.9	N/A
BMI (mean ± SD)	24.6 ± 3 .7	28.8 ± 5.6	0.072
Dyslipidemia (*n*; %)	0	15 (65)	0.0005^∗^
Hypertension (*n*; %)	0	17 (74)	0.0005^∗^
*Apolipoproteins(mean* ± *SD;(median; IQR 25 – 75) ng/ml)*			
Apo AI	175 ± 152 (121; 59-265)	421 ± 446 (235; 93-801)	0.08
Apo AII	75 ± 61 (59;24-120)	178 ± 191 (104; 40-293)	0.12
Apo CI	34 ± 21 (31; 14-50)	60 ± 49 (42; 22-108)	0.16
Apo CIII	1.5 ± 1.3 (1.2; 0.6-2.1)	4.0 ± 4.5 (2.2; 1.0-5.8)	0.053
Apo D	24 ± 10 (22; 17-29)	34 ± 17 (29; 17-50)	0.14
Apo E	72 ± 28 (75; 45-93)	95 ± 73 (79; 41-127)	0.54
Apo H	430 ± 243 (337; 288-586)	664 ± 539 (484; 275-943)	0.26
Apo J	401 ± 171 (384; 268-455)	640 ± 452 (564; 302-805)	0.11

^1^ Definition of healthy controls: no underlying disease, no medication.

## Data Availability

The data obtained in this study is available from the corresponding author upon request.
